# Extended reality to assess post-stroke manual dexterity: contrasts between the classic box and block test, immersive virtual reality with controllers, with hand-tracking, and mixed-reality tests

**DOI:** 10.1186/s12984-024-01332-x

**Published:** 2024-03-15

**Authors:** Gauthier Everard, Quentin Burton, Vincent Van de Sype, Thérèse Ntabuhashe Bibentyo, Edouard Auvinet, Martin Gareth Edwards, Charles Sebiyo Batcho, Thierry Lejeune

**Affiliations:** 1 Centre interdisciplinaire de recherche en réadaptation et intégration sociale, Université Laval, Québec, Canada; 2 Department of rehabilitation, Faculty of medicine, Laval University, Quebec, QC Canada; 3 Neuro Musculo Skeletal Lab (NMSK), Secteur des Sciences de la Santé, Institut de Recherche Expérimentale et Clinique, Université catholique de Louvain, Brussels, Belgium; 4 Service de médecine physique et réadaptation, Cliniques universitaires Saint-Luc, Avenue Hippocrate 10, Brussels, 1200 Belgium; 5 ECAM- Institut Supérieur Industriel, Brussels, Belgium; 6 Psychological Sciences Research Institute (IPSY), Université Catholique de Louvain, Louvain‑la‑Neuve, Belgium; 7 Louvain Bionics, Université catholique de Louvain, Louvain-la-Neuve, Belgium

**Keywords:** Stroke, Virtual reality, Augmented reality, Upper Extremity, Patient Outcome Assessment

## Abstract

**Background:**

Recent technological advancements present promising opportunities to enhance the frequency and objectivity of functional assessments, aligning with recent stroke rehabilitation guidelines. Within this framework, we designed and adapted different manual dexterity tests in extended reality (XR), using immersive virtual reality (VR) with controllers (BBT-VR-C), immersive VR with hand-tracking (BBT-VR-HT), and mixed-reality (MD-MR).

**Objective:**

This study primarily aimed to assess and compare the validity of the BBT-VR-C, BBT-VR-HT and MD-MR to assess post-stroke manual dexterity. Secondary objectives were to evaluate reliability, usability and to define arm kinematics measures.

**Methods:**

A sample of 21 healthy control participants (HCP) and 21 stroke individuals with hemiparesis (IHP) completed three trials of the traditional BBT, the BBT-VR-C, BBT-VR-HT and MD-MR. Content validity of the different tests were evaluated by asking five healthcare professionals to rate the difficulty of performing each test in comparison to the traditional BBT. Convergent validity was evaluated through correlations between the scores of the traditional BBT and the XR tests. Test-retest reliability was assessed through correlations between the second and third trial and usability was assessed using the System Usability Scale (SUS). Lastly, upper limb movement smoothness (SPARC) was compared between IHP and HCP for both BBT-VR test versions.

**Results:**

For content validity, healthcare professionals rated the BBT-VR-HT (0[0–1]) and BBT-MR (0[0–1]) as equally difficult to the traditional BBT, whereas they rated BBT-VR-C as more difficult than the traditional BBT (1[0–2]). For IHP convergent validity, the Pearson tests demonstrated larger correlations between the scores of BBT and BBT-VR-HT (*r* = 0.94;*p* < 0.001), and BBT and MD-MR (*r* = 0.95;*p* < 0.001) than BBT and BBT-VR-C (*r* = 0.65;*p* = 0.001). BBT-VR-HT and MD-MR usability were both rated as excellent, with median SUS scores of 83[57.5–91.3] and 83[53.8–92.5] respectively. Excellent reliability was found for the BBT-VR-C (ICC = 0.96;*p* < 0.001), BBT-VR-HT (ICC = 0.96;*p* < 0.001) and BBT-MR (ICC = 0.99;*p* < 0.001). The usability of the BBT-VR-C was rated as good with a median SUS of 70[43.8–83.8]. Upper limb movements of HCP were significantly smoother than for IHP when completing either the BBT-VR-C (t = 2.05;*p* = 0.043) and the BBT-VR-HT (t = 5.21;*p* < 0.001).

**Conclusion:**

The different XR manual tests are valid, short-term reliable and usable tools to assess post-stroke manual dexterity.

**Trial registration:**

https://clinicaltrials.gov/ct2/show/NCT04694833; Unique identifier: NCT04694833, Date of registration: 11/24/2020.

**Supplementary Information:**

The online version contains supplementary material available at 10.1186/s12984-024-01332-x.

## Background

Upper limb impairments are prevalent during both the acute [1, 2] and chronic phase [3] after a stroke. Such impairments result in activity limitations and participation restrictions, leading to a decline in the overall quality of life [4, 5]. In the field of neurorehabilitation, regular and time-bounded assessments of impairments and activity limitations are of utmost importance for establishing an effective rehabilitation plan [6, 7]. Moreover, functional assessments play a critical role in identifying prognostic factors that influence stroke recovery [8]. Recently, experts have formulated recommendations for the clinical evaluation of the upper limb in neurorehabilitation [9]. One such recommended assessment is the Box and Block Test (BBT) [10], which measures manual dexterity in the activity domain (according to the International Classification of Functioning, Disability and Health) [10]. Additionally, experts have highlighted the significance of kinematics in assessing body functions, through measures of movement quality and compensations during activity of daily living tasks [11].

In recent years, extended reality (XR) has emerged as an innovative and promising approach in rehabilitation. XR is a comprehensive concept that encompasses both present and forthcoming advancements in virtual reality (VR) and mixed reality (MR) [12]. VR technology allows for a computerized immersion of individuals in digitally created worlds, enabling them to experience multiple sensory stimuli and interact with the virtual environment through various modalities [13]. There are two primary types of VR experiences: non-immersive VR, where users remain aware of their physical surroundings and receive visual feedback through a 2D display, and immersive VR (iVR), which allows complete submersion in the virtual environment (using a Head Mounted Display (HMD) or a large curved screen with panoramic view) and provides a panoramic view [14]. More recently, MR systems have been developed [15] that offer individuals a hybrid experience by combining real objects and virtual environments to create a captivating midway point between these two realities [14]. MR can be classified as augmented reality and augmented virtuality systems. Augmented reality involves overlaying virtual information onto the physical environment whereas in augmented virtuality, real-world data is superimposed onto a virtual environment.

In the context of upper limb rehabilitation using XR, hand tracking and controllers are commonly employed as input devices to enable users to interact with the virtual environment [14, 16]. Hand-tracking technology measures hand position using HMDs equipped with infrared detectors or other specialized hardware (e.g., Leap Motion®), providing realistic visual feedback of hand and finger positions. However, current XR systems using hand-tracking technology do not allow for the provisioning of tactile feedback. On the other hand, controllers equipped with buttons and inertial measurement units enable the delivery of haptic feedback, but with limited visual feedback and positioning capabilities. Virtual representations of hand and fingers are not always available, and when they are, they do not always reflect natural hand positions. Most systems incorporating controllers tend to employ pre-determined hand poses that dynamically change based on the buttons pressed and the controller’s position, rather than accurately reflecting the real positions of hands and fingers. Both input methods (hand-tracking technology and controllers) have unique advantages and can be utilized based on the specific rehabilitation needs and goals of the individual.

Recently, VR has transcended its role as a therapeutic tool and has emerged as a valuable means of assessing upper limb body function [17], cognition [18, 19] and activities [20–22]. This assessment approach offers several advantages over traditional outcome measures. Firstly, VR allows for the implementation of computerized standardized protocols, effectively reducing the risk of inter and intra-rater bias. Secondly, it facilitates the measurement of multiple quantitative and objective variables, notably including reaction time, response time and kinematics, providing complementary performance measures that together build a more comprehensive understanding of the patient’s progress. Thirdly, once patients have been trained in VR assessment, they may gain the ability to conduct assessments independently, presenting the opportunity to increase the frequency of evaluations, even within the comfort of their homes.

Several iterations of the BBT have been developed in VR. Notably, two studies have demonstrated strong correlations between scores obtained in non-immersive VR versions and those of the traditional BBT [23, 24]. The first study was conducted among individuals with stroke [23] and the second among healthy participants and individuals with spinal cord injury [24]. Oña et al. took a step further by developing the first iVR BBT using an HMD and hand-tracking technology (Leap Motion®) to measure hand and finger movements [25]. Their study revealed a moderate correlation between the virtual BBT and traditional BBT scores among individuals with Parkinson Disease [25]. In a study by Dong et al., another immersive virtual BBT was designed, employing a specific haptic device [26]. The outcomes indicated moderate correlations between virtual and traditional versions of the test [26]. Similarly, we created an iVR BBT that employed controllers to manipulate and move the virtual blocks, while the HMD provided visual feedback [20]. Results demonstrated strong correlations between the number of blocks moved by individuals with stroke during the virtual and traditional BBT [20]. More recently, to further enhance the assessment process, a new manual dexterity test inspired by the BBT has been developed in MR using real blocks and an interactive non-immersive virtual environment display (REAtouch®, AXINESIS®, Belgium).

While several studies have explored the validity of VR-based BBT versions, a significant gap in the literature remains. To date, there have been no comprehensive investigations that directly compare different VR versions of BBT assessments, specifically contrasting responses made with hand-tracking vs. haptic devices. Hand-tracking technology may lead to improved sense of presence and more effective interaction when compared to haptic devices, but the accuracy and reliability of this technique remains debated [27]. Moreover, the validity of developing a manual dexterity test in MR remains under-explored. Yet, such developments could be of interest as, in contrast with hand-tracking and controller technologies, MR systems allow users to manipulate real word objects, therefore providing true haptic feedback. Addressing this research void is essential for gaining a comprehensive understanding of the relative advantages and limitations of these input modalities. Furthermore, to date, few studies have explored the potential of XR reality to assess upper limb kinematics. This study first aimed to bridge this gap by comparing the content and convergent validity of different XR BBT versions and manual dexterity tests using hand-tracking, controllers, and MR among healthy participants and individuals with stroke. We first hypothesized that scores from the iVR and MR tests versions would be strongly correlated to the traditional BBT. We also hypothesized that, on average, participants would displace more blocks in the traditional test and when using hand-tracking technology than when using controllers [27]. Secondary objectives were to assess and compare the reliability and usability of the different iVR and MR tests. Lastly, we aimed to compare upper limb kinematics that were acquired in iVR between individuals with stroke and healthy participants.

## Methods

### Study design

This prospective cross-sectional study was carried out in Belgium at the *Cliniques universitaires Saint-Luc* from October 2022 to September 2023. Ethical approval for the research was granted by the Saint-Luc-UCLouvain Hospital-Faculty Ethics Committee (reference 2015/10FEV/053). The experimental protocol was registered on clinicaltrials.gov (NCT04694833) and adheres to the Strengthening the Reporting of Observational Studies in Epidemiology (STROBE) guidelines. The supporting data for the findings of this study are outlined in Additional File 1.

### Participants

To achieve 80% power and a correlation coefficient (r) of 0.6 with a 5% significance level, a sample size of 20 stroke individuals with hemiparesis (IHP) was required. Inclusion criteria for individuals with stroke involved having experienced a first cortical or subcortical stroke episode with identifiable cerebral lesions according to World Health Organization criteria [28]. IHP were required to understand basic instructions and be able to move at least one cube from one side to another in the traditional BBT. Exclusion criteria included the presence of any other neurological or orthopaedic disorders that might impede upper limb movement, as well as insufficient visual acuity to read VR instructions. IHP were characterized in several ways: their initial stroke severity was determined using the National Institutes of Health Stroke Scale (NIHSS) obtained from their medical records [29]. The NIHSS score ranged from 0 to 42, with higher values indicating more severe neurological deficits. Upper limb motor control was evaluated using the computerized adaptive testing version of the Fugl-Meyer motor scale (CAT-FM), with scores converted to percentages using a Rasch model [30]. The testing conditions, scoring criteria, and instructions for the CAT-FM matched those of the traditional test. However, the CAT-FM employs an algorithm that allows for a potential reduction in the number of items. During the CAT-FM, examiners were prompted to report the score of each item to a web-based application. Based on the results of each item, the algorithm determined the subsequent item to be administered or decided whether there is adequate data to conclude the test without additional items. The overall score, reliability index and 95% confidence interval were calculated. Using Rasch analyses, the continuous data results were expressed in percentages, with higher percentages indicating better motor control. The CAT-FM was deemed valid and reliable to assess motor control among IHP [30]. Cognitive function was assessed using the Montreal Cognitive Assessment (MoCA) [31], with a score below 21/30 indicating cognitive impairment. The French version of the Modified Nottingham Sensory Assessment (EmNSA) was used to evaluate somatosensory function, with scores ranging from 0 to 44, where lower scores indicate greater somaesthetic impairment [32]. Healthy control participants (HCP) were included if they could comprehend basic instructions and had no neurological or orthopaedic disorders affecting upper limb function.

Healthcare professionals that were not members of our research team were also recruited. They were eligible to participate if they had at least two years of experience in rehabilitation and were familiar with using the BBT in clinical practice. All participants provided written informed consent before participating in the experiment.

### Material

For this experiment, different manual dexterity tests were used. This included the traditional BBT, two iVR versions of the BBT (one using controllers and the other using hand-tracking technology), and a MR test (inspired by the BBT). The order of administration was randomized.

The traditional BBT consisted of a wooden box measuring 53.7 cm x 24.4 cm x 8.5 cm, consisting of two compartments separated by a divider [10]. The objective was to move as many 2.5 cm wooden cubes as possible from one compartment to another using only one hand within 60 s, ensuring that the hand moving the block crossed over the partition. The score was determined by the number of correctly moved blocks within the allotted time.

**Fig. 1:**
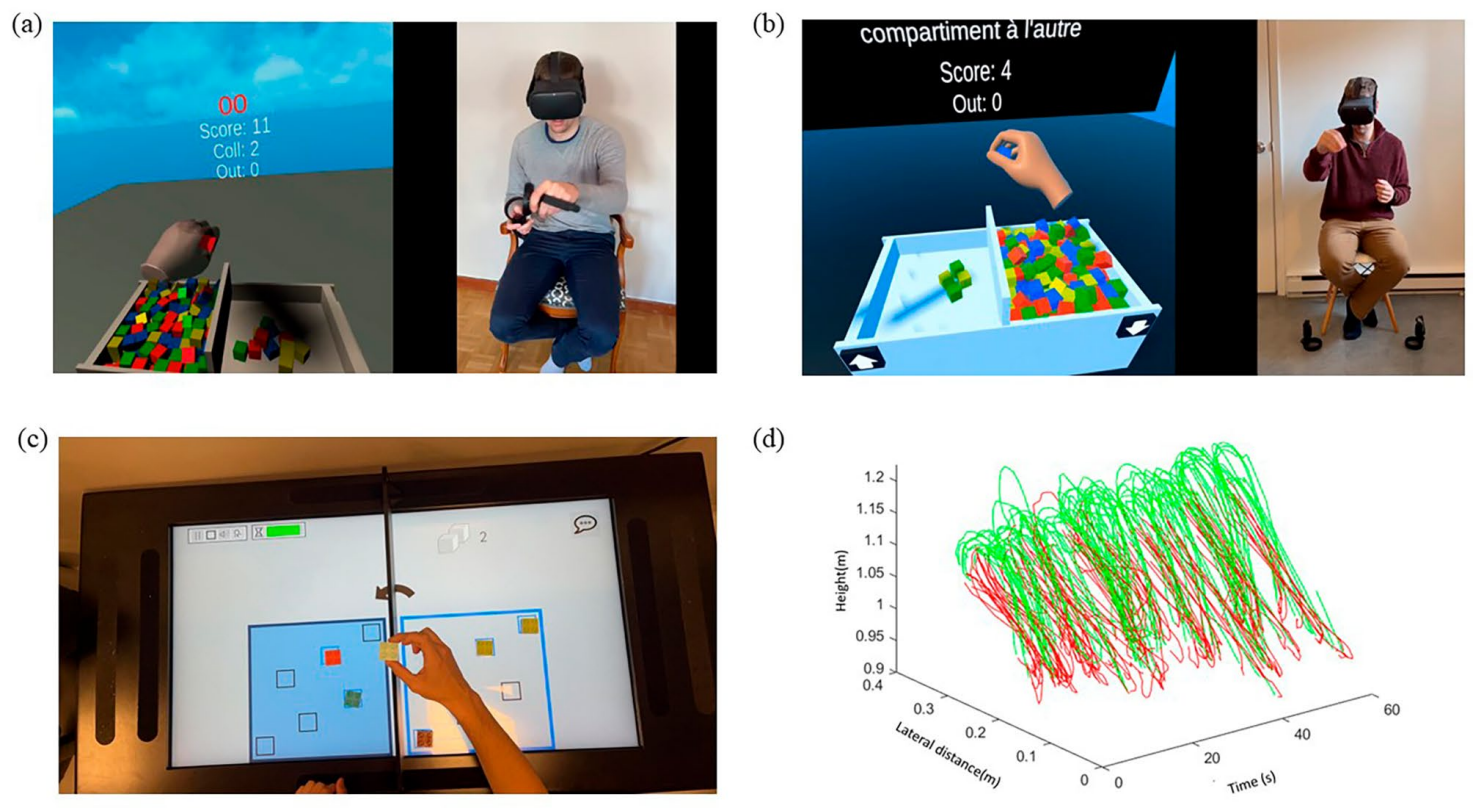
Representation of the BBT-VR-C, BBT-VR-HT and BBT-MR. This 4-panel figure simultaneously represents the virtual and mixed reality environment displayed in the systems and the movements performed by a healthy control subject to realize the task when interacting with the system. (**a**). This panel represent the BBT-VR-C. (**b**). This panel represents the BBT-VR-HT. (**c**). This panel represents the MD-MR. (**d**). This panel represents the evolution of controllers’ lateral and vertical position over a 60s trial of the BBT-VR-C. Motion executed by the hand from the moment of grasping a block to its successful release into the intended compartment within the virtual box are presented in green. Return movements to the compartment for the next block are presented in red

The two iVR versions were developed using Unity version 2021.3.22f1 (C# programming language) and utilized the Meta Quest 2^®^ standalone HMD (Meta^®^). For these two tests, dimensions of the virtual box and blocks were matched with these of the traditional BBT. The first iVR test, named BBT-VR-C (Fig. 1a), involved the use of controllers for block manipulation, whereby a virtual cube was grasped by pressing the controller’s buttons corresponding to the thumb-index or thumb-major or thumb-index-major grip. This test has been validated and was deemed reliable and usable for both HCP and IHP [20]. The second iVR test, named BBT-VR-HT (Fig. 1b), utilized hand-tracking technology, allowing participants to grasp and move virtual cubes based on their actual hand (finger and thumb) and arm movements. Such movement tracking is made possible thanks to 4 infrared cameras built into the front of the HMD, which constantly measure the positions and orientations of the individuals’ hands and fingers. A virtual cube can be grasped and lifted when the thumb and a long finger come into contact with the virtual cube, provided that the distance between the thumb and fingers corresponded to the size of the object. To release the cube, participants needed to open the aperture of their thumb and fingers or brought the thumb and fingers closer together.

The Manual Dexterity test in Mixed Reality (MD-MR) (Fig. 1c), was created by AXINESIS^®^ and involved use of an interactive tablet (REAtouch^®^). The REAtouch^®^ embeds up to 50 touch points in a 43-inch (95 × 53 cm) display to promote tactile and audiovisual interaction. The tablet is fully adaptable as it features a motorized height and tilt adjustment to cater to each patient’s needs and abilities. During the MD-MR, the interface was separated in two parts by a partition, whose height was similar to the traditional BBT. This partition was a standalone physical structure, akin to a wall separating the two boxes of the BBT, securely affixed to the REAtouch^®^ using Velcro, thereby ensuring stability during testing. Six plastic cubes were placed in predefined areas in the left or right side of the interface, depending on the side of the stroke. When the blocks contacted with the screen, they were detected by the REAtouch^®^ system. More specifically, throughout the test, the system identified whether the cubes were positioned correctly to calculate the score. Participants were asked to move the cubes to specific areas from one side to the other. Once this was done, they were asked to perform the same task, moving the cubes back in the initial side. This task was repeated over and over for a total duration of 60 s. As soon as the first block was moved, a timer started, and the number of blocks correctly moved was automatically calculated. The blocks had to be placed in one of the six dedicated areas (see Fig. 1c). The result was the number of blocks correctly moved during the allotted time.

### Procedure

To assess the content validity of the different XR manual dexterity tests, we assigned 5 therapists that were familiar with the BBT to perform the traditional BBT initially and then the 3 other XR tests in a random order. We asked them to first start with the traditional test to offer them a base of comparison when performing the other tests. After performing each test, therapists were required to respond to a questionnaire comprising items comparing their perceived difficulty to perform the tasks themselves in the given test relative to the traditional BBT. The following themes were covered by the different items of the questionnaire: the difficulty to open the hand when picking up the cube, the difficulty to grip the cube, the difficulty to maintain the grip throughout the movement, the difficulty to move the arm when displacing the cube, the difficulty to open the hand to release the cube, and the perceived tactile feedback (Additional file 1). For each question, the score ranged from − 2 (much easier with the test in question than with the BBT) to + 2 (much more difficult with the test in question than with the BBT). Answering 0 meant that the difficulty was the same between the 2 tests for the item concerned.

For the convergent validity, HCP and IHP were required to perform the BBT, the BBT-VR-C, the BBT-VR-HT and the MD-MR in a random order after receiving an explanation of the experimental protocol. All participants were assessed by the same investigator for each test. For the traditional BBT and MD-MR, the participant had to sit on a chair, with their feet on the floor, and with the test surface placed in front of them. For the BBT-VR-C and BBT-VR-HT, no surface was placed in front of them, and assessors could view the virtual environment via a streaming system displayed on a computer. The headset was placed by the same assessor each time, and participants were familiarized with the VR system. Drawing from our experience with XR, including IHP and the complexity of the systems, we decided to give participants 15 s of training for the BBT and MD-MR, and 30 s for BBT-VR-C and BBT-VR-HT. Then, each test was performed 3 times with the dominant hand for HCP and the paretic hand for IHP. These 3 trials aimed to minimize the learning effect. A rest period of 30 s was provided between each trial to limit the effect of fatigue on the performance. For the convergent validity analysis, we consistently considered score of the last trial only. The experiment lasted approximately 90 min.

To assess the usability of BBT-VR-C, BBT-VR-HT and MD-MR, all participants responded to the System Usability Scale (SUS) directly after performing the 3 trials of each test [33]. This questionnaire comprised 10 items and aimed to determine the subjective usability of the tested systems, using a Likert scale ranging from 0 to 100. A score between 52.01% and 72.80% indicated good usability, between 72.8% and 85.6% excellent usability, and between 85.6% and 100% best imaginable usability [33].

### Kinematics

For the BBT-VR-C and BBT-VR-HT, we conducted a kinematics analysis of hand positions using Visual Studio Code^®^ software (Python programming language). Initially, three-dimensional headset, controller (for the BBT-VR-C) and wrist (for the BBT-VR-HT) positions underwent spectral analysis to differentiate movement frequencies from background noise. Following this, we applied a 4th order Butterworth low-pass filter (sampling frequency = 72 Hz; cut-off frequency = 10 Hz) to minimize signal noise. To ensure the validity and reliability of the data, a visual analysis was performed by plotting hand positions over time (see Fig. 1d). Subsequently, we computed the kinematic path by taking the square root of the sum of squared positions. This path calculation allowed us to transform three-dimensional positions into dimensionless data, representing the distance covered by the hand in this study.

We further partitioned the path generated during one minute into distinct sub-movements. A sub-movement was defined as the motion executed by the hand from the moment of grasping a block to its successful release into the intended compartment within the virtual box. Therefore, our analysis exclusively considered the movement associated with transferring a block. As presented in Fig. 1d, we did not evaluate the return movement to the compartment for the next block.

For each participant’s sub-movement, we quantified hand motion quality through a movement smoothness index, the spectral arc length (SPARC), using the method outlined by Balasubramanian et al. [34]. SPARC is considered as the most valid kinematic index to quantify smoothness during reach-to-grasp and reach-to-point tasks [35]. SPARC represents the arc length of the Fourier magnitude spectrum of the velocity signal and has the advantage of being unaffected by movement amplitude and duration. This metric was analysed for the motion of each block throughout the 60-second trial, and average values were calculated.

### Statistical analyses

Statistical analyses were performed using SPSS version 29.0.1.0 (IBM^®^).

Content validity was first evaluated through interpretation of healthcare professionals’ median scores, along with the first and third quartiles, on the questionnaire.

For the convergent validity, we first checked the normal distribution of data using Shapiro-Wilk test. We then computed Pearson or Spearman correlations depending on linearity between the third trial’s score of each technological test (BBT-VR-C, BBT-VR-HT or MD-MR) and the third trial’s score of the BBT. We also computed Pearson or Spearman correlations between the third trial score of each XR test and the CAT-FM. Correlations were interpreted as small (0.1 < *r* ≤ 0.3), medium (0.3 < *r* < 0.5) or large (*r* ≥ 0.5) following Cohen’s recommendations [36]. These correlation coefficients were then compared using a Fisher z transformation [37].

We then conducted a repeated measures ANOVA and post-hoc tests to determine whether there were significant differences between the third score of the 4 tests. Each repeated measures ANOVA test was computed using Bonferroni or Tukey adjustments, depending on the normality of the data.

We also performed a repeated measure ANOVA and pairwise comparisons to evaluate the differences between the three trials of each test. We calculated test-retest reliability using a two-way mixed model Intraclass Correlation Coefficient (ICC) between the second and the third trial’s score of the 3 XR tests for HCP and IHP. We rated the reliability as poor (ICC or *r* ≤ 0.40), moderate (0.40 < ICC or *r* < 0.75) or excellent (ICC or *r* ≥ 0.75) [38].

Hand movement smoothness was compared between the third trial of BBT-VR-C and BBT-VR-HT conditions, and between IHP and HCP. To this end, we used a two-way ANOVA General Linear Model with the device (controllers vs. hand-tracking technology) and the population (IHP vs. HCP) as sources of variations. Holm-Sidak corrections were applied. Spearman or Pearson tests were used to assess correlations between SPARC and test scores in both BBT-VR-HT and BBT-VR-C conditions.

## Results

Twenty-one IHP (6 women) with a mean age of 65.7 ± 7.24 years old and 21 HCP (10 women) with a mean age of 56 ± 11 years old took part in the study. The IHP’s median time since stroke onset was of 3.2[0.58–5.72] months, the median upper extremity CAT-FM score was 77[77–96] %, the median MoCA score was 25[21.5–26.5], the median EmNSA score was 43[41.5–44], and the median NIHSS score of 8 [6–12] (Table 1; See Table 2 for the IHP individual information).

**Table 1:** Participants’ demographics

	Individuals with stroke (*n* = 21)	Healthy control participants (*n* = 21)
**Age (years)**	65 ± 7.2	56 ± 11.0
**Sex (F/M)**	6/15	10/11
**Dominant hand (L/R)**	2/19	2/19
**Side of stroke (L/R)**	13/8	/
**Time since stroke onset (months)**	3.2 [0.58–5.72]	/
**Upper extremity CAT-FM (%)**	77 [77–96]	/
**EmNSA (/44)**	43 [41.5–44.0]	/
**MoCA (/33)**	25 [21.5–26.5]	/
**NIHSS (/42)**	8 [6–12]	/

F = Female; M = Male; L = Left; R = Right; CAT-FM = Computerized adaptive version of the Fugl-Meyer; EmNSA = French Version of the Modified Nottingham Sensory Assessment; MoCA = Montreal Cognitive Assessment; NIHSS = National Institute of Health Stroke Scale

**Table 2:** Individuals with stroke’s characterization

Subject	Age (years)	Paretic hand	Hand dominance	Time since stroke onset (days)	Lesion location	NIHSS	MoCA	EmNSA
**1**	62	Right	Right	352	Spontaneous left thalamo-capsular haemorrhagic stroke	16	20	8
**2**	68	Left	Right	145	Right fronto-parietal haemorrhagic stroke	8	26	44
**3**	78	Left	Right	31	Ischaemic stroke of the right internal capsule	6	23	43
**4**	75	Right	Right	11	Left frontal ischaemic stroke	6	25	44
**5**	62	Left	Right	36	Right internal capsular ischaemic stroke	6	27	43
**6**	68	Left	Right	180	Right sided sylvian ischaemic stroke	3	26	43
**7**	63	Left	Right	1044	Right anterior ischaemic stroke	12	23	26
**8**	69	Right	Right	108	Left thalamic ischaemic stroke	9	23	43
**9**	65	Right	Right	18	Low posterior ischaemic stroke of the left internal capsule	7	25	43
**10**	63	Right	Right	19	Ischaemic stroke of the crown radiata & left pontic region	4	17	44
**11**	55	Left	Right	140	Right sylvian haemorrhagic stroke	18	26	41
**12**	55	Left	Right	863	Haemorrhagic stroke right thalamus	10	26	42
**13**	74	Right	Right	312	Left frontal ischaemic stroke	18	12	32
**14**	53	Left	Right	218	Right internal capsular ischaemic stroke	10	24	44
**15**	57	Right	Right	10	Left internal capsule stroke	5	28	42
**16**	75	Left	Right	134	Right sided sylvian ischaemic stroke	9	27	44
**17**	67	Left	Right	83	Right capsulo-thalamic haemorrhagic stroke	8	20	44
**18**	69	Left	Right	13	Right anterior ischaemic stroke	4	13	44
**19**	59	Left	Left	17	Right-sided sylvian ischaemic stroke	15	23	33
**20**	72	Left	Left	146	Right internal capsular ischaemic stroke	/	/	44
**21**	71	Right	Right	17	Left-sided sylvian ischaemic stroke	/	/	43

NIHSS = National Institute of Health Stroke Scale; MoCA = Montreal Cognitive Assessment; EmNSA = French Version of the Modified Nottingham Sensory Assessment

Five healthcare professionals participated in the content validity study. They had a mean age of 27 ± 1.3 years old, and a mean of 2.7 ± 0.84 years of experience using the BBT to evaluate IHP’s manual dexterity. At the time, they had experienced VR on themselves an average of 4 ± 3.8 times, and they experienced using VR on IHP an average of 5 ± 8.7 times.

### Primary outcome - content and convergent validity

For content validity, healthcare professionals provided a median score of 0[0–1] for both the BBT-VR-HT and MD-MR, indicating their perception that the movements required to perform the tests were, on average, as difficult as in traditional BBT. However, they rated the movements involved in the BBT-VR-C as more difficult than those involved in the traditional BBT (1[0–2]). For the BBT-VR-C, the difficulty to open the hand when picking up a cube (1[0–2]), to move the arm when displacing the cube (1[0–1]) and to open the hand to release the cube (1[0–2]) were rated as higher than when performing the traditional BBT. The lack of tactile feedback was considered as a major complication of the test (2 [1–2]). For the BBT-VR-HT, the closing of the hand when gripping the cube was rated as more difficult than the traditional BBT (1[0–1.5]), and the lack of tactile feedback was considered as a limitation of the test (2 [1–2]). Finally, for the MD-MR, arm movements required to move the cube (1[0–2]) were rated as more difficult than the traditional BBT. Complementary information can be found in Table 3.

**Table 3:** Content validity results

Item	BBT-VR-C	BBT-VR-HT	MD-MR
Opening the hand when picking up the cube	1 [0–2]	0 [0–1.5]	0 [0–1]
Closing the hand when gripping the cube	0 [-0.5–2]	1 [0–1.5]	0 [0–0.5]
Maintaining the grip throughout the movement	0 [-1–1]	0 [-0.5–1]	0 [0–0]
Arm movement to displace the cube	1 [0–1]	0 [0–0]	1 [0–2]
Opening of the hand to release cube	1 [0–2]	0 [0–1.5]	0 [0–1.5]
Tactile feedback	2 [1–2]	2 [1–2]	0 [0–0.5]

Content validity data. Range for each item goes from − 2, meaning the task is much easier to do with the concerned test than with traditional BBT, to + 2, meaning the task is much more difficult to do with the concerned test than with traditional BBT.

**Fig. 2:**
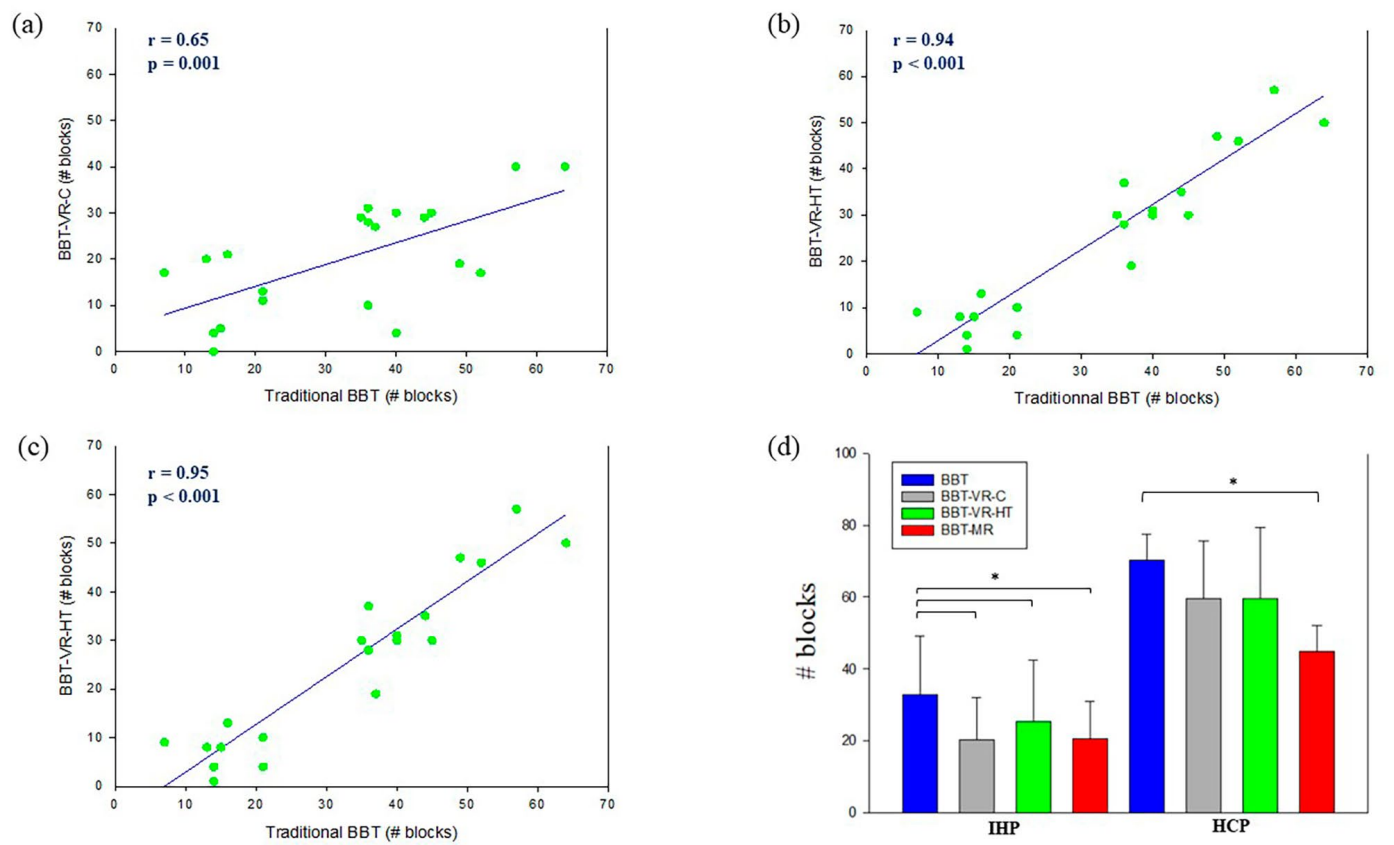
Correlations and score differences between BBT, BBT-VR-C, BBT-VR-HT and MD-MR. (**a**). In this correlation plot, each point represents paretic hand’s score obtained when performing the BBT in relation to the BBT-VR-HT score. Pearson correlation coefficients (r) and their *p*-value (*p*) are presented at the left side of the graph. A linear regression is plotted in blue. (**b**). In this correlation plot, each point represents paretic hand’s score obtained when performing the BBT in relation to the BBT-VR-HT score. Pearson correlation coefficients (r) and their *p*-value (*p*) are presented at the left side of the graph. A linear regression is plotted in blue. (**c**). In this correlation plot, each point represents paretic hand’s score obtained when performing the BBT in relation to the MD-MR score. Pearson correlation coefficients (r) and their *p*-value (*p*) are presented at the left side of the graph. A linear regression is plotted in blue. (**d**). In this plot, HCP and IHP’ mean BBT, BBT-VR-C, BBT-VR-HT and MD-MR scores are presented as histograms with error bars. Each error bar represents the positive standard deviation of the given test’s score. The * corresponds to a significant difference

For convergent validity with IHP, Pearson tests demonstrated large correlations between the scores of BBT and BBT-VR-C (*r* = 0.65; *p* = 0.001; Fig. 2a), BBT and BBT-VR-HT (*r* = 0.94; *p* < 0.001; Fig. 2b), and BBT and MD-MR (*r* = 0.95; *p* < 0.001; Fig. 2c). The correlation observed between BBT and BBT-VR-HT scores was larger than the correlation between BBT and BBT-VR-C scores (Fisher z=-8.0; *p* < 0.001). Similarly, the correlation between BBT and MD-MR scores was larger than the comparison between BBT and BBT-VR-C scores (Fisher z=-4.0; *p* < 0.001). Repeated measures ANOVA (F = 15.3; df = 20; *p* < 0.001) and post-hoc tests revealed that the score of the traditional BBT (33 ± 16.2) was significantly higher than these of the BBT-VR-C (20 ± 11.8; t = 5.9; *p* < 0.001), BBT-VR-HT (25 ± 17.0; t = 3.5; *p* = 0.005) and MD-MR (21 ± 10.3; t = 5.8; *p* < 0.001) (Fig. 2d).

Spearman tests demonstrated no correlation between the scores of CAT-FM and BBT-VR-C (*r* = 0.35; *p* = 0.113), a medium correlation between the scores of CAT-FM and BBT-VR-HT (*r* = 0.45. *p* = 0.038) and a large correlation between the scores of CAT-FM and MD-MR (*r* = 0.72; *p* < 0.001). The correlation between CAT-FM and MD-MR scores was significantly larger than the correlation between CAT-FM and BBT-VR-C scores (Fisher z = 2.5; *p* = 0.006), and the correlation between CAT-FM and BBT-VR-HT scores (Fisher z = 2.6; *p* = 0.005).

For HCP, Friedman repeated measures ANOVA on rank test (χ²=25.5; df = 3; *p* < 0.001) and pairwise comparisons revealed that the score of the traditional BBT (70[66.0–75.0]) was significantly higher than the MD-MR (46[42.5–48.5]; q = 7; *p* < 0.001), but not significantly different than the BBT-VR-C (61[45.5–71.0]; q = 3.2; *p* = 0.105), and BBT-VR-HT (57[46.0–79.0]; q = 2.9; *p* = 176) (Fig. 2d).

### Secondary outcomes – test-retest reliability

For IHP, repeated measures ANOVA and post-hoc tests (Table 4) revealed that there were consistent significant score increases between the first and third trial of the BBT (t = 6.3; *p* < 0.001), BBT-VR-C (t = 3.4; *p* = 0.004), BBT-VR-HT (t = 4.0; *p* < 0.001) and MD-MR (t = 3.4; *p* = 0.005). There was no significant difference between the score of the first and second trial, nor between the score of the second and third trial for any of the tests. We therefore measured associations between the second and third trial score of each test. Excellent reliability was found for the BBT-VR-C (ICC = 0.96; *p* < 0.001), BBT-VR-HT (ICC = 0.96; *p* < 0.001) and MD-MR (ICC = 0.99; *p* < 0.001).

**Table 4:** Test-retest reliability results

	Trial 1	Trial 2	Trial 3	ICC (2vs3)	*p*-value (ICC)	*p*-value (ANOVA or Friedman) *
IHP						
BBT-VR-C	17 ± 8.6	18 ± 11.1	20 ± 11.8	0.96	< 0.001	0.005
BBT-VR-HT	20 ± 12.5	23 ± 15.5	25 ± 17.0	0.96	< 0.001	0.001
MD-MR	19 ± 10.3	20 ± 9.8	21 ± 10.3	0.99	< 0.001	0.007
HCP						
BBT-VR-C	46 ± 15.9	54 ± 17.1	60 ± 16.2	0.92	< 0.001	< 0.001
BBT-VR-HT	52 ± 19.8	60 ± 21.8	60 ± 19.8	0.86	< 0.001	0.005
MD-MR	42 [39–45.5]	43 [42–47.5]	46 [42.5–48.5]	0.76	< 0.001	0.072

ICC = Intraclass Correlation Coefficient; *Bonferroni adjustments applied for ANOVA post-hoc tests and Tukey adjustments applied for Friedman ANOVA pairwise comparisons; IHP = Individuals with stroke suffering from hemiparesis; HCP = Healthy control participants

For HCP, repeated measures ANOVA and post-hoc tests revealed that there was a significant score increase between all the trials of the BBT-VR-C (1vs2: t = 4;2, *p* < 0.001; 2vs3: t = 3.0, *p* = 0.012 ;1vs3: t = 7.3, *p* < 0.001), and between the first and second trials (t = 3.1; *p* = 0.001), and first and third trials (t = 2.9; *p* = 0.016) of the BBT-VR-HT. Friedman repeated measures ANOVA on rank test revealed no significant score differences between the three trials of the MD-MR (χ²=5.3; df = 2; *p* = 0.07). Excellent reliability was found for the BBT-VR-C (ICC = 0.92; *p* < 0.001), BBT-VR-HT (ICC = 0.86; *p* < 0.001) and MD-MR (ICC = 0.76; *p* < 0.001) when measuring associations between the scores of the second and third trials.

### Secondary outcomes – usability

**Fig. 3:**
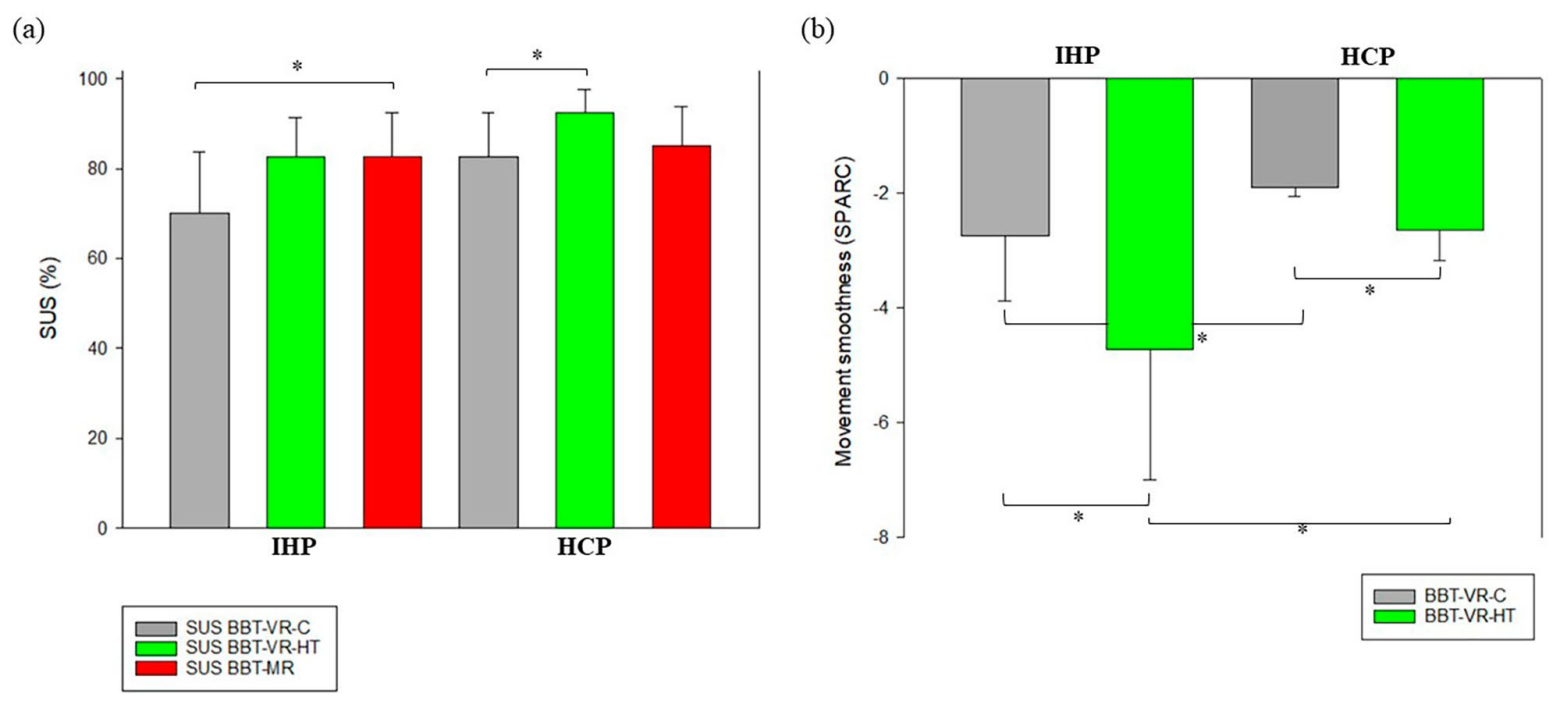
Differences in usability score and upper limb kinematics. (**a**). In this plot, HCP and IHP’s median of BBT-VR-C, BBT-VR-HT and MD-MR SUS scores are presented as histograms with error bars. Each error bar represents the 3rd quartile of the given test’s score. The * corresponds to a significant difference. (**b**). In this plot, HCP and IHP’s mean movement smoothness, as measured by the spectral arc length, are presented in the form histograms with error bars. Each error bar represents the positive standard deviation of the given test’s score. The * corresponds to a significant difference

For IHP, the BBT-VR-C usability was rated as good with a median SUS of 70[43.8–83.8]. Usability of BBT-VR-HT and MD-MR were both rated as excellent, with median SUS scores of 83[57.5–91.3] and 83[53.8–92.5] respectively. Friedman repeated measures ANOVA on rank test (χ²=8.9; df = 2; *p* = 0.012) and pairwise comparisons revealed that the SUS of the MD-MR was significantly greater than this of the BBT-VR-C (q = 3.7; *p* = 0.024). There were no significant differences between the SUS of the BBT-VR-HT and the SUS of the BBT-VR-C (q = 3.2; *p* = 0.06) and between the SUS of the BBT-VR-HT and this of the MD-MR (q = 0.5; *p* = 0.921) (Fig. 3a).

HCP gave a median SUS score of 83[67.5–92.5] for the BBT-VR-C, 93[82.5–97.5] for the BBT-VR-HT and 85[76.3–93.8] for the MD-MR, providing excellent usability. Friedman repeated measures ANOVA on rank test (χ²=8.1; df = 2; *p* = 0.017) and pairwise comparisons revealed that the SUS of the BBT-VR-HT was significantly greater than this of the BBT-VR-C (q = 3.8; *p* = 0.019). There were no significant differences between the SUS of the BBT-VR-HT and the SUS of the MD-MR (q = 2.4; *p* = 0.206), or between the SUS of the BBT-VR-C and this of the MD-MR (q = 1.4; *p* = 0.575) (Fig. 3a).

### Secondary outcomes – upper limb kinematics

As presented in Fig. 3b, upper limb movements of HCP (SPARC_BBT−VR−C_=-1.9[-1.99–-1.79]; SPARC_BBT−VR−HT_=-2.6[-2.95–-2.24]) were significantly smoother than these of IHP (SPARC_BBT−VR−C_=-2.3[-3.02–-2.03]; SPARC_BBT−VR−HT_=-3.6[-6.01–-3.21]) when completing either the BBT-VR-C (t = 2.05; *p* = 0.043) or the BBT-VR-HT (t = 5.21; *p* < 0.001). When comparing the iVR tests conditions, we observed that IHP (t = 4.83; *p* < 0.001) exhibited smoother upper limb movements when passing the BBT-VR-C than when passing the BBT-VR-HT but not HCP (t = 1.85; *p* = 0.068). Moreover, Spearman tests showed that SPARC and each VR test score were strongly correlated for the BBT-VR-HT (*r* = 0.74; *p* < 0.001) and the BBT-VR-C (*r* = 0.73; *p* < 0.001).

## Discussion

This study first aimed to assess and compare the validity of different post-stroke manual dexterity XR tests provided in iVR with controllers, iVR with hand-tracking, and MR. The IHP showed strong correlation of results on the traditional BBT scores compared to iVR and MR tests, supporting their convergent validity. Nevertheless, iVR and MR tests scores were notably 30% lower than these of the traditional BBT. The short-term test–retest reliability was deemed excellent for both IHP and HCP across all three XR tests. IHP rated BBT’s usability as excellent when using iVR hand tracking and MR, and as good when using the controller. Kinematic analyses revealed that HCP performed smoother upper limb movements than IHP when completing the iVR tests.

### Comparison with previous studies

Several XR versions of the BBT and other technological hand mobility tests have recently emerged to improve assessment of manual dexterity in neurorehabilitation [23–26, 39]. However, to our knowledge, few studies have explored the content validity of these adaptations, leaving a critical gap in understanding their usefulness. While most studies have shown moderate-to-strong correlations between scores of XR tests and traditional BBT, some variability persists depending on the system used and the population assessed. For instance, our results, although generally aligning with previous research [23–26, 39], revealed higher convergent validity in IHP for manual dexterity tests involving MR or hand-tracking iVR technology compared to systems using iVR controllers. These findings contrast Oña et al. who used a hand-tracking iVR technology among individuals with Parkinson disease, but only found a moderate correlation between the scores of the traditional BBT and their iVR version [25]. This deviation from our findings could stem from the fact their participants had a more limited range of scores on the traditional BBT, spanning from 30 to 66. Yet, a restricted range of scores may weaken correlations [40]. Interestingly, our results well align with the findings of Molla-Casanova et al., (2021) who developed a hand mobility assessment using a digital tablet, but without object manipulation. They found large correlations between most scores provided by their test and those of the BBT, the Fugl-Meyer, the Jebsen Taylor-Hand Function Test and the Nine Hole Peg Test [39].

Regarding secondary outcomes, in line with our results, three prior studies have examined the short-term reliability of their virtual BBT versions using controllers, hand-tracking, and a haptic device in iVR, all consistently finding excellent reliability [20, 25, 26]. Another team found an excellent reliability using a tablet-based hand mobility test [39]. While our results may suggest that different interactions modes in iVR are all reliable, further protocols might be of interest to identify how these interactions modes in addition to other co-variates such as the age, affinity for technology, severity and type of motor, sensitive and cognitive impairments, affect the reliability of manual dexterity assessment in IHP when using these new technologies. In line with our prior study, we also observed that reliability results between IHP and HCP were relatively similar for the BBT-VR-C [20]. However, for the BBT-VR-HT and the MD-MR, we found better reliability results for IHP than for HCP. One plausible explanation could be attributed to the narrower ranges and dispersion indexes of manual dexterity scores for trials 2 and 3 by HCP compared to those of IHP. This difference in inter-subject variability is known to weaken the ICC correlations, although this argument holds true only for the MD-MR, as higher ranges and dispersion indexes are observed for HCP in the other tests. Another factor to consider is that the reliability of BBT-VR-HT and MD-MR might be more robust for low-score performances. This suggests that the consistency of measurements is particularly notable in situations where manual dexterity is initially limited, providing insights into reliability dynamics across different performance levels.

In terms of usability, our findings align with a limited number of studies. In Oña et al., individuals with Parkinson’s disease and their healthcare providers rated usability as high to excellent based on a satisfaction questionnaire [25]. In our first study, IHP rated the usability of BBT-VR-C as good (79%) on the SUS [20].

### Hand-tracking vs. controllers

In the current study, the usability assessments of the VR using hand tracking and controllers closely matched for IHP whereas, among HCP, usability was rated as higher when using hand tracking over controllers. Nevertheless, the debate over which input method offers optimal usability in iVR remains an ongoing topic of discussion. In fact, some studies suggest that both controller and hand-tracking systems offer similar ease of use when training medical students in procedures like intubation [41], while younger healthy subjects tend to prefer controllers over hand-tracking for object manipulation or gaming in VR [42, 43].

Both controllers and hand-tracking technologies in iVR present distinct advantages and drawbacks. Controllers allow for leveraging inertial measurement units and infra-red tracking, offering precise interaction in the virtual environment, mimicking the sensation of object interaction for users and enhancing overall immersion. However, the manipulation of controllers may fall short of replicating natural hand movements, potentially compromising the realism and validity of assessments.

Conversely, hand-tracking technology eliminates the need for physical controllers, allowing users to interact freely with the virtual world using their hands and fingers directly. This approach enhances immersion and facilitates natural interaction, as users can manipulate virtual objects without pressing any buttons. Hand-tracking proves advantages for intuitive gestures and movements, fostering a more fluid and user-friendly experience. However, a potential drawback is the current limitations in tracking precision and haptic feedback [44]. Fine-grained interactions may pose challenges, and users might miss the tactile feedback offered by physical controllers. Additionally, hand-tracking may encounter difficulties in scenarios involving complex hand movements or when the hands are out of the tracking field.

### Immersive virtual environment vs. real environment

A substantial finding in our study was the notable difference in block-moving performance between iVR versions and the traditional BBT. This discrepancy can be attributed to multiple factors. First, the perception of distance and depth of field, often underestimated in iVR [45] due to the technology’s inherent limitations, can significantly impact task performance [46]. These limitations include narrower fields of view [47, 48], HMD weight [48], and geometric distortions [49] that may result in misjudgments during block manipulation tasks.

Second, the very nature of immersion in a virtual environment introduces complexity, as VR experiences inherently differ from real-world encounters. This contrast can particularly affect individuals less familiar with using emerging technologies [50], compromising their ability to fully engage and execute precise movements. Findings from several studies indicate that there may be individuals who respond differently to technological interventions, suggesting the presence of both responders and non-responders within stroke population [51, 52]. This may further contribute to the score differential observed between the traditional BBT and the MD-XR. Nevertheless, despite this score difference, a recent study has demonstrated no disparity in motor cortex activations between virtual and traditional BBT conditions, probably suggesting that the immersion of VR does not affect the sensorimotor control [53].

Third, the lack of realistic tactile feedback in VR systems employing controllers and hand-tracking potentially diminishes the immersion factor, impacting the participant’s sense of presence within the virtual environment. The integration of multisensory feedback has been proven to significantly enhance reaction times in tasks completion [54]. Our study and feedback from participants also revealed challenges in using the controller, particularly related to button-controlled hand movements. The physical attributes of controllers, including weight and size, can exacerbate difficulties for individuals with upper limb impairments. The situation may become even more complex for those with cognitive impairments, as it can impede their ability to comprehend controller manipulation. However, it is worth mentioning that we were unable to validate this hypothesis due to the relatively high MoCA scores in our sample.

### Mixed reality vs. real environments

Our findings underlined a disparity in scores between the traditional BBT and the MD-MR, which can be primarily attributed to the precision and oculo-manual coordination demands of the MR task that involves precise cube manipulation and placement. In this assessment, participants were tasked with bidirectional meticulous and exact placements of cubes, which inevitably translated to slower movement speeds in comparison to the conventional BBT, where participants can rely on a less precise toss of the block cube into the box. This discrepancy is even more accentuated among HCP and is likely a consequence of their need to strike a balance between speed and precision of their movements. These observations resonate with existing scientific literature, reinforcing the understanding that fine motor precision tasks inherently lead to reduced movement speed [55]. These insights also underscore that the MD-MR’s deviation from the traditional BBT could be essentially attributed to the precision component.

Without this requirement for precision, the MD-MR resembles the traditional BBT in terms of overall scoring. The MD-MR demonstrates superior content and convergent validity compared to the other tests, affirming its considerable potential for clinical integration.

### Upper limb kinematics

The finding that upper limb movements in HCP were significantly smoother than those of IHP during iVR tests is consistent with prior research indicating that HCP tend to exhibit more fluid movements in virtual environments [56, 57]. This aligns with the general understanding that motor deficits in individuals with stroke can lead to less smooth and coordinated movements compared to healthy individuals, as highlighted in studies exploring motor control and kinematics post-stroke [57, 58].

The fact that both IHP and HCP displayed smoother movements during the BBT-VR-C compared to the BBT-VR-HT is also in line with previous work emphasizing the influence of VR system characteristics, such as hand-tracking technology and sensory feedback, on movement kinematics [41].

### Implications

The different tests presented in this paper demonstrate notable validity, usability, and reliability, establishing their significance as valuable tools applicable in both clinical and research settings. These applications offer several advantages that can facilitate their adoption. First, the use of iVR and platforms allows for the collection and analysis of kinematic data, aligning with clinical recommendations for assessing upper limb impairment in neurorehabilitation [6]. Notably, the hand-tracking system, given its alignment with natural hand and finger movements, is increasingly becoming a subject of study and validation in this context [44]. These technological and kinematic assessments may contribute to a deeper understanding of individuals who continue to enhance their quality of movement or compensate even after reaching a plateau in block-moving capabilities. Second, these XR tools have the potential to increase the frequency of functional assessments, aligning with recent best practice guidelines [6, 59]. Following a training period, individuals with sufficient motor and cognitive abilities could independently perform the test, even from their homes, enhancing user satisfaction [60]. Additionally, the cost of the VR headsets becomes increasingly more accessible. The latest headsets have the advantage of not requiring a connection to a computer and automatically receiving updates to enhance features such as hand tracking.

In terms of research implications, on the one hand, the equivalent score between the BBT-VR-C and BBT-VR-HT could suggest that the lack of tactile feedback in iVR may not significantly impact manual dexterity performance. However, the challenges posed by the complexity of both iVR tests may be sufficiently important to attenuate or even void the effect of providing tactile feedback during the test. On the other hand, the observed superior usability and validity of MD-MR compared to BBT-VR-C might reflect the importance of providing realistic tactile feedback to ensure a positive user-experience and a certain sustainability. These aspects should be considered when developing technological assessments for individuals with varying comprehension and cognitive abilities within the IHP population. It might be worthwhile to explore the integration of instrumental gloves [61] or more physiologically adapted controllers in upcoming studies.

### Limits and perspectives

This study presents several limitations. First, while the comparison of the MD-MR with other versions of the BBT provided valuable insights, it should be noted that the MD-MR’s specific operational mode not only emphasizes speed and precision, but also requires manual manipulation in two directions, making it different from the traditional BBT. To enable a more direct comparison, future studies could explore a system that eliminates the need for precision in the task, closely mimicking the traditional BBT. Second, the familiarization period was more important for the iVR tests than for the BBT and MR tests due to their greater complexity. However, BBT-VR-HT and BBT-MR both showed excellent usability and were found to be equally difficult. Future studies might investigate how participants familiarize with the different XR systems. Third, test-retest reliability was only examined in the short-term, within the same session. Further investigations should explore mid-term reliability, assessing the consistency of results over the days following the first assessment. Similarly, the sensitivity to change could be studied within a larger population to better understand how these tools perform in various clinical contexts. Fourth, while the comparison of arm kinematics between HCP and IHP was primarily focused on assessing hand movement smoothness through the SPARC index, the employment of hand-tracking technology opens up possibilities for more detailed analyses. This could include examining velocity and accuracy indexes, as well as exploring the interaction between hand and finger movements. Moreover, the kinematics analysis was only made for the tests developed in iVR, as the traditional BBT does not involve any technology and the MD-MR does not yet provide arm kinematics information. Exploring the use of inertial measurements units and marker less cameras could be valuable to compare arm kinematics in the four test conditions. Lastly, given its potential effect on performance and perceived usability of virtual systems, the age disparity between HCP and IHP may have influenced outcomes of kinematics comparison. Future research could address this by more precisely matching age (and other relevant characteristics) between these groups.

## Conclusion

The BBT-VR-C, the BBT-VR-HT and the MD-MR are valid, short-term reliable and usable tools to assess post-stroke manual dexterity. The BBT-VR-C and BBT-VR-HT provide kinematic data that allow for the measurement of smoothness outcomes. All these tests hold potential to be used both in research and clinical practice.

### Electronic supplementary material

Below is the link to the electronic supplementary material.

Supplementary Material 1

## Data Availability

The dataset supporting the conclusions of this article is included within the article (and its additional file(s)).

## Abbreviations

BBT: Box and Block Test
XR: Extended Reality
VR: Virtual Reality
MR: Mixed Reality
iVR: Immersive Virtual Reality
HMD: Head Mounted Display
STROBE: Strengthening The Reporting for Observational studies in Epidemiology
IHP: Individuals with troke suffering from HemiParesis
HCP: Healthy Control Participants
NIHSS: National Institutes of Health Stroke Scale
CAT: FM–Computerized Adaptive Testing of the Fugl–Meyer
EmNSA: The French version of the Modified Nottingham Sensory Assessment
MoCA: Montreal Cognitive Assessment
BBT–VR–C: Virtual reality version of the Box and Block Test involving the use of Controllers
BBT-VR–HT: Virtual Reality version of the Box and Block Test involving the use of Hand–Tracking
MD-MR: Manual Dexterity Test in Mixed Reality
SPARC: Spectral Arc Length
ICC: Intraclass Correlation Coefficient
